# Editorial: Mechanisms of Novel Drugs and Gene Modifiers in the Treatment of Cystic Fibrosis

**DOI:** 10.3389/fmolb.2022.975946

**Published:** 2022-07-12

**Authors:** Guido Veit, Iris Silva, Massimo Conese, Onofrio Laselva

**Affiliations:** ^1^ Department of Physiology, McGill University, Montréal, QC, Canada; ^2^ S2AQUAcoLAB, Olhao, Portugal; ^3^ Department of Clinical and Experimental Medicine, University of Foggia, Foggia, Italy

**Keywords:** CFTR, cystic fibrosis, CFTR corrector, SLC6A14, IGFBP-6

## Introduction

Cystic fibrosis (CF), one of the most common hereditary diseases, is caused by mutations in the CF Transmembrane Conductance Regulator (CFTR) gene. More than 2000 mutations with varying clinical severity are known, which result in a range of cellular phenotypes, categorized into six classes characterized by expression, folding, gating, conductance, quantity and peripheral stability defects, respectively (Veit et al., 2016). In recent years CFTR modulators that improve the folding (correctors) and gating (potentiators) have been developed and compounds that increase read-through of premature termination codons, inhibit nonsense-mediated decay, stabilize the CFTR mRNA, inhibit aberrant splicing and prevent premature degradation of the mutant channel are being investigate (Cao et al., 2020; Oren et al., 2021; Venturini et al., 2021; Kim et al., 2022). Other lines of research aim to ameliorate the pathophysiological manifestations of CF by restoring mucociliary clearance, by attenuating the hyperinflammation, or by activating alternative ion conductance pathways (Laselva et al., 2022). Understanding the molecular and physiological mechanisms of these approaches is prerequisite for their successful implementation. In this special Research Topic we collected five studies that use different perspectives providing mechanistic insights into approaches that change the pathophysiology of CF. In particular, three original articles examined the therapeutical approaches to increase the mutated CFTR function by small molecules and two original articles investigated the role of other transporters (*i.e.* SLC6A14) or a novel mediator (*i.e.* IGFBP-6) in CF airway inflammation.

## Approaches targeting mutant CFTR

To date, three combination therapies (Orkambi™ [VX-809+VX-770], Symdeko™ [VX-661+VX-770] and Trikafta™ [VX-445+ VX-661+VX-770]) have been approved for CF patients bearing the *F508del* mutation on at least one allele (Cuevas-Ocana et al., 2020). It has been demonstrated that prolonged exposure of bronchial epithelial cells with the CFTR potentiator VX-770, reduced VX-809-mediated F508del-CFTR correction (Cholon et al., 2014; Veit et al., 2014). Interestingly, prolonged (15 days) treatment of bronchial epithelial cells with the first-generation CFTR corrector VX-809 resulted in the dedifferentiation of epithelium-like polarized cell monolayers, which was prevented by co-treatment with hepatocyte growth factor (HGF) (Matos et al., 2018). In the current manuscript Matos at al. demonstrated that the second-generation CFTR corrector (VX-661) does not dedifferentiate the polarized bronchial epithelial monolayers. Moreover, HGF restored the Symdeko rescue of F508del-CFTR function in bronchial epithelial cells in the presence of VX-770.

Since HGF inhibits amiloride-sensitive epithelial Na^+^ channel (ENaC) function and protects from the deleterious effect of VX-770 on F508del-CFTR function, co-treatment of HGF with CFTR modulator combinations could improve the clinical responses of CF patients to the therapy.

However, there are still 5–10% of CF patients bearing premature termination codons (PTC) mutations, who will not directly benefit from those therapies. Fang et al. tested the efficacy of CFTR modulators in combination with readthrough compounds (i.e. the aminoglycoside antibiotic G418) to rescue the most common PTC CF-causing mutation in the remaining 10% of patients. The authors showed that the G542X-CFTR protein expression was rescued only using the combination of C1 corrector, which binds to N-terminal of CFTR protein, and G418 but not with other CFTR corrector types. Therefore, they demonstrated that the use of efficacious read-through reagents is the prerequisite for mitigating the G542X-CFTR deficit.

The peripheral protein quality control system subjects plasma membrane (PM) localized CFTR to a second level of quality control, ubiquitinates misfolded proteins and targets these to lysosomal degradation. This results in attenuated PM residence time and reduced channel function of misfolded CFTR and may attenuate modulator efficacy. In their current manuscript Taniguchi et al. identified the E3 ubiquitin ligase RNF34 as a mediator of misfolded F508del-CFTR peripheral quality control. Dual knockdown of RNF34 and the previously identified E3 ubiquitin ligase RFFL (Okiyoneda et al., 2018) resulted in increased PM expression and function of F508del-CFTR, which was additive to the Trikafta™ modulator drug combination. Thus, RNF34 and RFFL are potential drug targets that may increase the efficacy of Trikafta™ therapy.

## Approaches targeting the pathophysiological manifestations of CF

Modifier genes induce phenotype variability between people with CF carrying the same CFTR mutations. Among these, the gene encoding for the amino acid transporter SLC6A14 has been recently studied and associated with lung disease severity and age of primary airway infection by the bacteria *Pseudomonas aeruginosa*. In this study, Mercier et al. investigated the functional effect of rs3788766, located within SLC6A14 promoter. The authors showed that in the presence of the rs3788766 minor allele G the individuals with CF in a large French cohort had a more severe lung disease. By using a luciferase reporter assay the authors showed that the SLC6A14 promoter activity was reduced in the presence of the G allele, and a decrease in SLC6A14 activity was associated with decreased mTOR phosphorylation, as well as bronchial epithelial repair rates in wound healing assays. This suggests that SLC6A14 might influence CF lung phenotype via the mTOR signaling pathway and epithelial repair process modulation.

The discovery of novel pathophysiologic biomarkers in CF is mandatory to guide therapeutic interventions. Insulin-like growth factor binding protein 6 (IGFBP-6) has been recently shown to play a putative role in the innate immune system, however its expression and regulation at the level of the airway epithelium is not known yet. Laselva et al. found higher basal expression of IGFBP-6 cells bearing the *F508del* mutation compared to WT-CFTR. Moreover, the IGFBP-6 expression was increased in both WT- and F508del- CFTR CFBE and in patient-derived nasal epithelial cultures (HNE) cells under infection and inflammatory conditions. Dymethil fumarate, a FDA-approved drug with anti-inflammatory and anti-oxidant properties, reduced IGFBP-6 levels as induced by lipopolysaccharide (LPS). Interestingly, IGFBP-6 decreased pro-inflammatory cytokines expression in a dose-dependent fashion while not altering Trikafta™-dependent F508del-CFTR functional expression. These data suggest IGFBP-6 may represent a novel mediator in CF airway inflammation, and that is druggable.

We expected that this Research topic will contribute to developing novel strategies to increase the efficacy of CFTR modulators and to obtaining better understanding the pathophysiology of CF lung disease.
